# A Single-Chip CMOS Pulse Oximeter with On-Chip Lock-In Detection

**DOI:** 10.3390/s150717076

**Published:** 2015-07-14

**Authors:** Diwei He, Stephen P. Morgan, Dimitrios Trachanis, Jan van Hese, Dimitris Drogoudis, Franco Fummi, Francesco Stefanni, Valerio Guarnieri, Barrie R. Hayes-Gill

**Affiliations:** 1 Electrical System and Optics Research Division, Faculty of Engineering, University of Nottingham, University Park, Nottingham NG7 2RD, UK; E-Mails: diwei.he@nottingham.ac.uk (D.H.); steve.morgan@nottingham.ac.uk (S.P.M.); 2 Keysight Technologies Belgium NV, Kortrijksesteenweg 1093 B, 9051 Sint Denijs Westrem, Belgium; E-Mails: dimitrios.trachanis@keysight.com (D.T.); jan.vanhese@keysight.com (J.V.H.); Dimitris.Drogoudis@cirrus.com (D.D.); 3 EDALab-Networked Embedded Systems, Ca Vignal, 2-Strada Le Grazie, 15-37134 Verona, Italy; E-Mails: franco.fummi@edalab.it (F.F.); francesco.stefanni@edalab.it (F.S.); valerio.guarnieri@edalab.it (V.G.)

**Keywords:** pulse oximetry, SpO2, photoplethysmography, heart rate monitor, smart sensor, lock-in, CMOS sensor

## Abstract

Pulse oximetry is a noninvasive and continuous method for monitoring the blood oxygen saturation level. This paper presents the design and testing of a single-chip pulse oximeter fabricated in a 0.35 µm CMOS process. The chip includes photodiode, transimpedance amplifier, analogue band-pass filters, analogue-to-digital converters, digital signal processor and LED timing control. The experimentally measured AC and DC characteristics of individual circuits including the DC output voltage of the transimpedance amplifier, transimpedance gain of the transimpedance amplifier, and the central frequency and bandwidth of the analogue band-pass filters, show a good match (within 1%) with the circuit simulations. With modulated light source and integrated lock-in detection the sensor effectively suppresses the interference from ambient light and 1/f noise. In a breath hold and release experiment the single chip sensor demonstrates consistent and comparable performance to commercial pulse oximetry devices with a mean of 1.2% difference. The single-chip sensor enables a compact and robust design solution that offers a route towards wearable devices for health monitoring.

## 1. Introduction

The blood oxygen saturation level indicates the percentage of hemoglobin molecules in the arterial blood which are saturated with oxygen, and has been identified as an indicator of risk in chronic diseases of the circulatory and respiratory system. Pulse oximetry was introduced in 1983 as a noninvasive method for monitoring the arterial oxygen saturation of a patient’s blood [1]. Oxygen saturation (SpO2) is defined as the measurement of the amount of oxygen dissolved in blood, based on the detection of oxygenated hemoglobin (HbO_2_) and deoxygenated hemoglobin (Hb).

(1) $$\text{SpO}2=\;\frac{{\text{HbO}}_{2}}{{\text{HbO}}_{2}+\text{Hb}}\times 100\%$$

Two different light wavelengths are used to obtain the SpO2 value. The absorption of HbO_2_ and Hb differs greatly at the first wavelength, and is approximately equal at the second wavelength. Typically 660 nm (red) and 940 nm (infrared) wavelengths are used [1].

The signal obtained from a single wavelength is called the Photoplethysmogram (PPG), which is used for heart rate monitoring [2]. It consists of a small pulsatile component due to the light absorption by arterial blood, and a large static component due to light absorption by bone, tissue, skin, hair and venous blood. The dual wavelength pulse oximeter calculates the absorbance ratio (R) which indicates the magnitude of SpO2 and is given by Equation (2):

(2) $$R=\;\frac{I_{ac,Red}/I_{DC,Red}}{I_{ac,IR}/I_{DC,IR}}$$

where the symbols *I_ac_* and *I_DC_* denote the ac component and dc component of the red and infrared (IR) PPG signals respectively. Typically *R* ranges from 0.5 to 2 [1] and is a useful measurement as it is independent of the absolute light intensity of the LED and the optical properties of the tissue without arterial blood. It is then compared to look up tables that are based on a calibration curve developed empirically from various known SpO2 levels to relate *R* to oxygen saturation.

There are two main configurations of pulse oximetry namely “transmission” or “reflectance” [3,4]. In transmission mode, the device is placed peripherally on a finger, toe, or earlobe where light is transmitted through the tissue having pulsatile blood flow and is detected on the other side of the tissue. In reflectance mode, the photodetector and light source are both on the same side. The reflectance pulse oximeter allows measurements to be taken from areas of the body in which transmittance based pulse oximetry cannot be applied, making it more suitable for ambulatory monitoring such as the abdomen and forehead [5].

Recently miniature designs of the reflectance pulse oximeter have been developed for wearable health monitoring. Haahr *et al.* [6] designed an electronic patch with a size of 88 mm by 60 mm and Li and Warren’s design [7] is 41 mm by 36 mm. Huang *et al.* [8] developed a ring-type optical probe with an external circuit board of approximately 40 mm by 20 mm. Recent development of pulse oximeters also includes interfacing a conventional clinical oximeter finger sensor to smart-phone devices [9]. For developing a small, robust and low cost device a single chip design is an attractive solution. However, the reported work [10,11,12,13,14] on the chip level designs of a pulse oximeter and the PPG focus on low power circuits with the systems operating in transmission mode. For example the designs reported in [10,11,12] are purely analogue (except for the LED timing circuit) and the chip outputs are analogue PPG or SpO2 signal. In [13] an external analogue to digital converter (ADC) and a PC are used for digitization and signal processing whilst [14] also reports off-chip digital signal processing. Moreover, all of these chip designs require an external photodiode for light detection.

This paper describes a single-chip pulse oximeter consisting of photodiode, transimpedance amplifier, analogue band-pass filters, analogue-to-digital converters, digital signal processor (including lock-in) and LED timing control. The novelty of the design lies in the fully integrated circuit implementation which enables a compact, robust and low cost design solution providing the potential for it to be used as a wearable device for health monitoring. The device can be operated in reflection or transmission geometry and incorporates on chip modulated LED illumination drive circuitry and integrated lock-in detection so that the sensor effectively suppresses the ambient light and 1/f noise of the transimpedance amplifier.

The paper is broken down into Section 2 that describes the design and system configuration of the sensor. Section 3 describes the experimental set up and methods deployed to characterize the CMOS sensor. Section 4 describes the experimental results of the analogue circuit characterization, *in vivo* measurements for the complete analogue and digital CMOS design where the results are compared with commercial CE marked and FDA approved devices. Discussion and conclusions follow in Section 5.

## 2. Sensor Design and PCB Design

**Figure 1:**
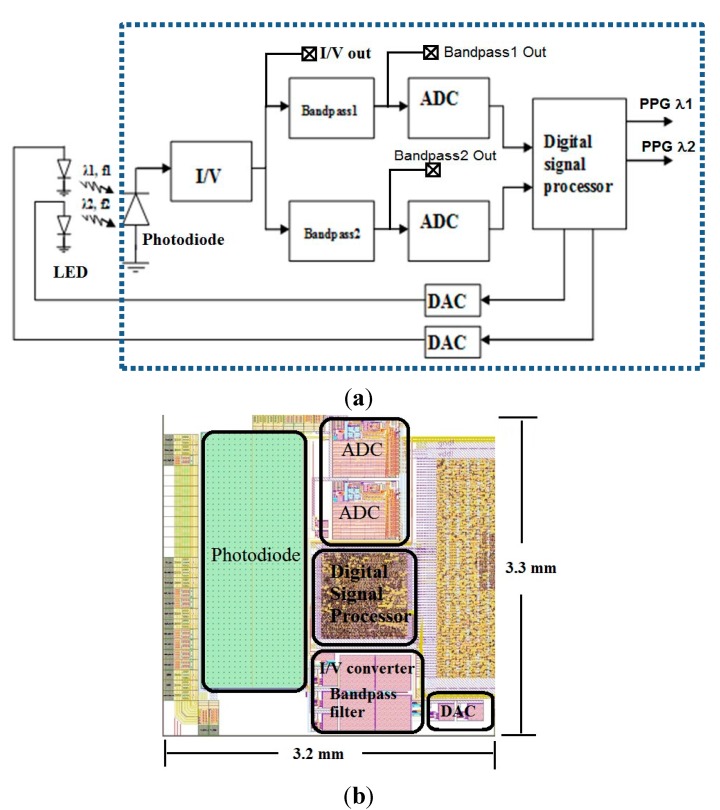
(**a**) Block diagram of the sensor. (Photodiode, I/V—Current to Voltage Converter, ADC—analogue to digital converter, Digital signal processor, DAC—digital to analogue converter, LED—light emitting diode)—Items inside dotted region are all “CMOS on-chip”; (**b**) Layout of the CMOS sensor.

Figure 1a illustrates a block diagram of the pulse oximeter sensor. It consists of a 1 mm × 2.5 mm n-well-p-substrate photodiode, a linear transimpedance amplifier having an 8 M Ohm feedback resistor and 30 KHz bandwidth, two Sallen-Key band-pass filters having 2 KHz (band-pass1) and 4 KHz (band-pass2) filter bandwidths respectively, two 10-bit successive approximation register (SAR) ADCs running at a sampling rate of 640 KHz. The digital signal processor includes a quadrature demodulator and LED timing control unit generating 10 KHz and 20 KHz square waves. The LEDs shown in the figure are off-chip components whilst all other components illustrated inside the dotted line are all integrated into a single semi-custom CMOS chip.

Light sources with two different wavelengths (λ1 = 660 nm, λ2 = 940 nm) are modulated with square waves at 10 KHz and 20 KHz respectively to enable frequency division multiplexing and to eliminate the effects of ambient light on the detected signal. These frequencies are used because they are unlikely to be present within the operating environment and are above the 1/f noise corner frequency (~1 KHz) of the I/V converter. The light backscattered by the skin is detected by the photodiode, which is of size 1 mm × 2.5 mm and is an n-well-p-substrate type common in CMOS structures for junction isolation [15]. The current to voltage converter is based on an operational amplifier and has a linear response to the generated photocurrent. It has an 8 M Ohm feedback resistor and a bandwidth of 30 KHz, which is above the modulation frequencies and therefore it is sufficient for detecting the 10 KHz and 20 KHz channels corresponding to the two wavelengths. The output dynamic range of the current to voltage converter is between 1.65 V to 3.17 V, and can be observed off chip through a buffer for more detailed sub-circuit characterization. The subsequent band-pass filters are of type Sallen-Key having 2 KHz (band-pass1) and 4 KHz (band-pass2) filter bandwidths centred at the modulation frequencies (10 KHz and 20 KHz respectively). These filters allow additional isolation of the two carriers (λ1 = 660 nm, λ2 = 940 nm), provide anti-alias filtering, and attenuate interfering constant and low frequency light sources at the photodiode. The output of the band-pass filters is then sampled by the 10-bit successive approximation register (SAR) ADCs running at a sampling rate of 640 KHz [16]. The SAR ADC utilises synchronous clock generation and has differential input. For chip characterization, output signals can be taken at intermediate points in the circuit (after the transimpedance amplifier and the band pass filters). The digitized data are demodulated by the on chip digital signal processor using a quadrature demodulator (lock-in) to recover the PPG signal [17]. The demodulator multiplies digitized data by local oscillator (LO) signals that have a 90 degree phase shift between them to generate in phase and quadrature (I and Q) channels. The root-mean-square (RMS) combination of I and Q channels provides the demodulated PPG signal. The demodulator processes 64 raw data values in real-time for the 10 KHz channel (and 32 raw data values for the 20 KHz channel), and outputs a single value for subsequent accumulation. Therefore in total it stores only 96 10-bit raw data values. As multiplications and accumulations are involved, the PPG output expands to 24-bit. The quadrature demodulation algorithm was firstly modelled in Matlab, then written in VHDL and simulated in ModelSim. The layout of analogue and digital circuits was generated in Virtuoso and Encounter (Cadence) respectively. The digital processing unit consists of 4139 standard logic gates and consumes 4.6 mW power over a 1 mm × 1 mm silicon area.

Mixed-signal simulation has been carried out to validate the system design. The VerilogA models of analogue circuits and the VerilogAMS model of the ADC were simulated in Advanced Design System (Keysight Technologies, Belgium). SystemVue (Keysight Technologies) captured the output, together with the VHDL simulation of ModelSim, to run a mixed-signal simulation for the whole system. For a simulated time of 1 s, an execution time of 30 min was needed on a PC (Windows 7, i7 2.7 GHz CPU, 16 GB RAM). In order to accelerate the simulation speed, the VerilogA and VerilogAMS models were translated into native SystemVue models, and the VHDL code was translated into C++ models by HIF Suite (EDALab) and imported into SystemVue. The execution time was reduced to 8 min.

The chip layout shown in Figure 1b is of size 3.2 mm × 3.3 mm and was fabricated in a 0.35 µm four metal layer CMOS process (Austriamicrosystems). Regions of the chip other than the photodiodes were covered by a top (4th) metal layer to prevent illuminating parts of the chip that are not intended to be light sensitive.

**Figure 2:**
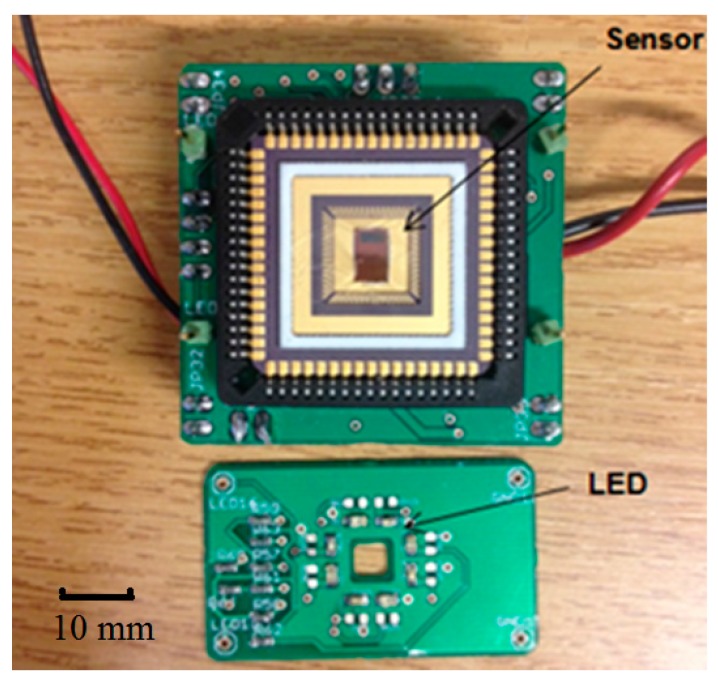
CMOS Sensor chip and LED illumination PCBs.

Figure 2 shows the prototype including a test board with the CMOS sensor chip and an LED board. The light source consists of four λ = 660 nm (ROHM—SML-310LTT86N) and four λ = 940 nm (KINGBRIGHT—KP-2012F3C) LEDs, providing even illumination to the tissue. There are four holes at the corners of the LED board and four corresponding pins on the CMOS sensor board for fixation. In reflection configuration, the LED board is placed at the top of the CMOS sensor board with LEDs facing upward, the light backscattered by the tissue passes through the central hole (4 mm × 4 mm) and illuminates the photodiode on the CMOS sensor. The separation of the LED and the CMOS sensor is 5 mm. The system can also work in transmission mode by flipping the LED board so that it is situated on top of the finger.

## 3. Test Methodology

### 3.1. Chip Characterization

**Figure 3:**
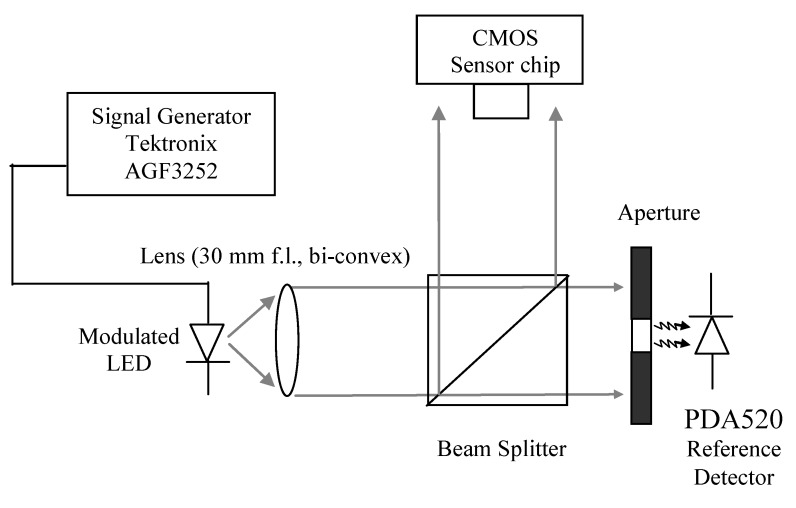
Optical setup for laboratory characterization of the I/V converter and band-pass filters. The ac and dc light levels of the λ = 640 nm red LED (KINGBRIGHT—L-7104SRC-J4) is controlled by a signal generator (Tektronix AGF3252). The reference photodiode (PDA520) has a known transimpedance gain and is used to calculate the light falling on the CMOS sensor.

AC and DC laboratory test characterization of the I/V converter and band-pass filters were carried out. The experimental setup shown in Figure 3 uses a λ = 640 nm LED (KINGBRIGHT—L-7104SRC-J4) to produce the optical test signal. The intensity and modulation depth of the light are controlled by a signal generator (Tektronix AGF3252). The light is collimated by a 30 mm focal length bi-convex lens and passes through a beam-splitter which enables illumination of both the pulse oximetry sensor and a reference photodiode (PDA520, Thorlabs). The on chip 1 mm × 2.5 mm photodiode is an n-well-p-substrate type whose responsivity is approximately 0.3 A/W at a wavelength of 667 nm [15]. Table 1 shows the light power incident on the photodiode, the corresponding generated photocurrent and the voltage output of the transimpedance amplifier. It should be noted that the dark current output is 1.65 V representing the effective analogue ground in the circuit. The reference photodetector has known transimpedance gain of 10^6^ V/A and is used to calculate the amount of light falling onto the sensor.

**Table 1:** Table illustrating the photodiode illuminance, corresponding photocurrent and resulting transimpedance (I/V) amplifier output voltage. Note: the output voltage for dark current is 1.65 V.

Light Power (nW)	Photocurrent (nA)	Voltage (V)
20.83	6.25	1.7
135.83	43.75	2.0
354.17	106.25	2.5
633.33	190	3.17

The output voltages of the I/V converter, band-pass filters and the reference photodetector are then recorded by a digital oscilloscope (Tektronix TDS2004C). As the transimpedance gain of the reference photodetector is known, the reference photocurrent can be used to obtain the AC and DC photocurrents generated at the CMOS sensor after appropriate scaling for detector area.

### 3.2. In Vitro Pulse Oximetry Experiments

As discussed in Section 1, all pulse oximeters require calibration to relate the absorbance ratio (R) to SpO2. To perform *in vitro* calibration, three translucent artificial fingers (BC biomedical FingerSim) are used which contain dyes simulating arterial blood at 80%, 90% and 97% oxygen saturation levels across the red and near infrared wavelength range. The FingerSim can only be used in transmission mode. The designed CMOS pulse oximeter was therefore used to measure the three R values from the three artificial finger phantoms having known SpO2. By plotting R ratio *versus* SpO2 a calibration curve can then be constructed which allows R values to be related to SpO2.

### 3.3. In Vivo Pulse Oximetry Experiments and Comparison with Commercial Devices

*In vivo* experiments are carried out in either reflection or transmission geometry using the design shown in Figure 2 with a finger placed on top of the LEDs. In reflectance, backscattered light passes through the hole at the centre of the LED board (housing the λ = 660 nm and 940 nm LEDs) and strikes the sensor. Simultaneous measurements of SpO2 were carried out in order to compare the results with those of commercial devices. The designed CMOS sensor, a Massimo Radical-7 and a Mind Media Nexus-10 simultaneously measure the SpO2 levels of the middle finger, index finger and ring finger respectively. The volunteer was asked to hold the breath for a period of 50 s so as to generate a desaturation event. Studies in healthy participants using this protocol have been approved by the Faculty of Engineering Ethics committee at the University of Nottingham.

## 4. Results

### 4.1. CMOS Chip Characterization

**Figure 4:**
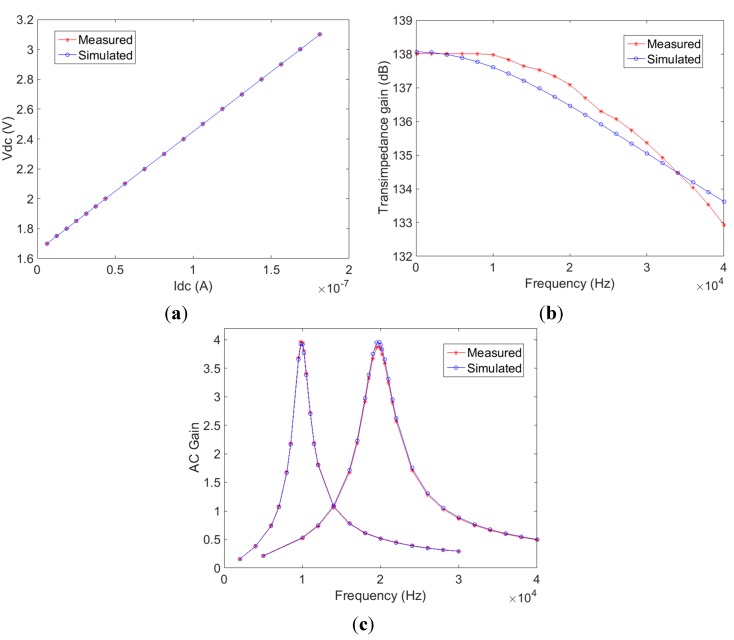
Experimental and simulated chip characterization results (**a**) DC response of the I/V converter; (**b**) Frequency response of the I/V converter; (**c**) Frequency response of the two band-pass filters.

Figure 4a shows the measured DC output voltage of the op-amp I/V converter as the DC photocurrent is varied. As expected the output voltage increases linearly as the dc photocurrent increased until the output saturates at 3.1 V. Figure 4b shows the measured frequency response of the I/V converter at a typical current generated in the *in vivo* experiments (240 nA DC photocurrent). At low frequencies the transimpedance gain is 8 MΩ due to the 8 MΩ feedback resistor and the circuit has a −3 dB cut-off frequency at 30 KHz. By observing the ac amplitudes of the I/V converter and the two band-pass filters, the voltage gains of the band-pass filters are calculated and shown in Figure 4c. The band-pass filters have centre frequencies at 10 KHz and 19.9 KHz with −3 dB bandwidth of 2 KHz and 4 KHz respectively. The measured results including Vdc output of the transimpedance amplifier, transimpedance gain of the transimpedance amplifier, and the central frequency and bandwidth of the analogue band-pass filters are also illustrated by way of comparison in Figure 4 with Cadence simulation. It was found that the measured results, indicate <1% difference to the original design specification (as simulated).

The gain of the band-pass filter is calculated by dividing the measured amplitude of the band-pass filter output by the transimpedance amplifier output. The small gain difference between the two channels is likely due to process variation.

### 4.2. In Vitro Pulse Oximetry Experiments

**Figure 5:**
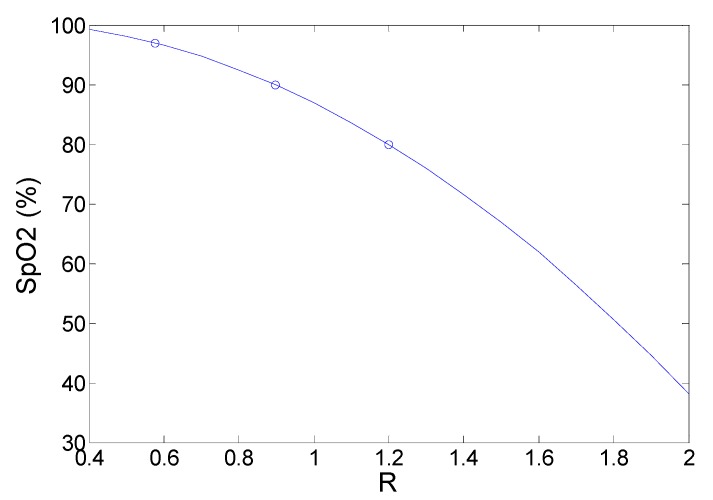
Second-order empirical calibration curve relating the absorbance ratio (R) from the CMOS sensor to the SpO2 level of the “BC biomedical FingerSim” phantoms in *transmission* mode.

Deployment results of the full CMOS sensor *in vitro* to measure the equivalent SpO2 of the “BC biomedical FingerSim” phantoms in *transmission* geometry are shown in Figure 5. The measured R ratio values from the CMOS sensor for 97.5%, 90% and 80% SpO2 phantoms were 0.58, 0.92 and 1.15 respectively. To allow the ratio R to be related to the oxygen saturation over a wide range, a second order polynomial curve calculated from Matlab is used:

SpO2 = 100.5 − 4.15 × R − 17.69 × R^2^ (3)

This is the method utilized in most commercial pulse oximeters [1].

### 4.3. In Vivo Pulse Oximetry Experiments and Comparison with Commercial Devices

**Figure 6:**
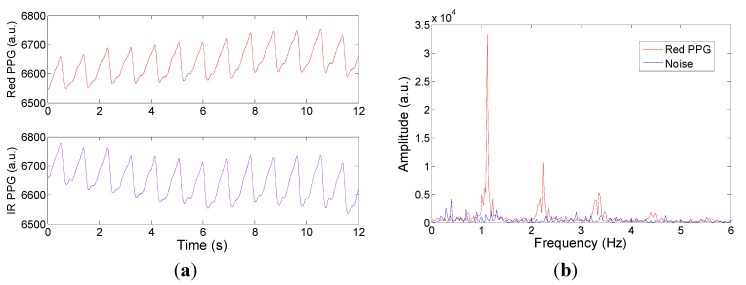
(**a**) Red λ = 660 nm (top) and IR λ = 940 nm (bottom) PPG signals taken in *reflectance* mode from the finger of a healthy volunteer; (**b**) Frequency spectrum of the Red PPG signal (DC removed) and system noise floor.

The CMOS sensor was then applied *in vivo* to a healthy volunteer in both transmission and reflectance mode. After CMOS on chip digital demodulation, typical red and IR PPG signals can be captured and displayed. Figure 6a shows the typical output PPG signal obtained in reflectance mode with the frequency spectrum of the red channel plotted in Figure 6b from which a heart rate of 1.1 Hz (~66 bpm) can be clearly observed with associated harmonics. The noise floor obtained from a static tissue phantom is also plotted for comparison.

**Figure 7:**
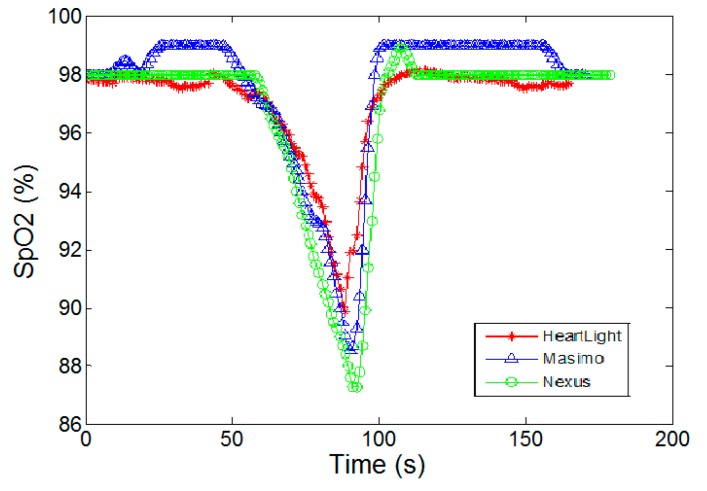
Comparison of the SpO2 values for the CMOS sensor and two commercial devices (Masimo Radical 7 and Nexus-10) in *transmission* mode for a breath hold and release experiment.

Figure 7 shows a comparison of the CMOS sensor chip (HeartLight) with two CE marked & FDA approved commercial pulse oximeters (Masimo Radical 7 and Nexus-10) in *transmission* geometry during a “breath hold and release” experiment. Using Equation (3) the SpO2 values of the CMOS sensor were determined from the measured R ratio values. Figure 7 shows that the output of the CMOS sensor is generally in agreement with the two commercial SpO2 devices. It can be seen that the oxygen saturation decreases from 98% to 88% during the breath holding period and then increases sharply back to 98% and the CMOS sensor chip faithfully follows this trend.

**Figure 8:**
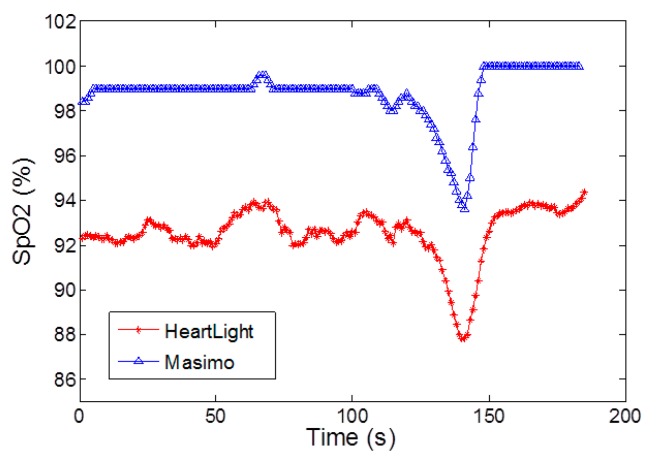
Comparison of the SpO2 outputs of the CMOS sensor and a Masimo Radical-7 in *reflectance* mode for a breath hold and release experiment.

A comparison is also made between the CMOS sensor chip and the Masimo Radical 7 in *reflectance* mode in Figure 8 using a breath hold and release experiment. It should be noted that the Nexus-10 can only be used in transmission mode and hence the data is not available. In addition as the finger simulator is not valid in reflection geometry then it was not possible to generate an accurate calibration curve. However, for direct comparison illustrating the experimental trend, rather than an accurate quantification, Equation (3) was still applied. The results of Figure 8 illustrate that the CMOS sensor output generally reads 6% lower than the Masimo Radical-7 output but nevertheless they show a good agreement in terms of the timing and trend of the % of increase/decrease during the breath hold and release experiment.

## 5. Discussion and Conclusions

A single-chip CMOS pulse oximeter has been designed, fabricated and tested. The CMOS chip consists of photodiode, I/V converter, analogue band-pass filters, ADCs, digital signal processor (with digital lock-in detection) and LED timing control. The I/V converter and band-pass filters were optically and electrically characterised and the measured results compare well with the design specifications (<1% difference). The modulated light sources and integrated digital lock-in detection enables the sensor to effectively suppress interference from ambient light and 1/f noise. PPG signals using 660 nm and 940 nm wavelengths are presented and an empirical calibration curve for transmission geometry was derived using commercially available dye based translucent phantom SpO2 fingers.

During *in vivo* breath-holding experiments, changes in SpO2 can be observed and the trends and absolute values show good agreement with two commercial devices namely the Masimo Radical 7 and the Nexus-10 in transmission mode. In reflection geometry however, the phantom fingers are not valid and hence the calibration curve of the CMOS sensor possesses an offset when compared with the commercially calibrated reflectance mode device (Masimo Radical 7). This latter point will be addressed in future by comparing the reflectance measurements with realistic blood samples and blood gas analysis.

The overall packaging is relatively large (25 mm × 25 mm) as it uses a JLCC68 packaging for test purposes. In subsequent prototypes, quad flat no-leads (QFN) or chip-scale packaging can be employed in order to miniaturize the device footprint. This will reduce the overall size to 5 mm × 5 mm. Future versions of the chip will also investigate a fully integrated chip in which the SpO2 calibration curve is implemented on-chip along with a heart rate extraction algorithm.
